# 

**Published:** 1846-03

**Authors:** 


### INDEX.

PAGE

Abstract, Half-Yearly, of the Medical Sciences. By W. H. Ranking, M.D. 116
Acupuncture, case of singultis, of several years standing, cured by. By Dr. Emiliani, - - - - - 16
Adulteration of Saffron, - - - - - 80
American Quarterly Journal of Agriculture and Science, - 89
An Address on Insanity, and the establishment of a Lunatic Asylum, delivered before the Committee of the House of Representatives, on Education and the Public, Dec. 25, 1843. By John Evans, M.D., of Attica, Ia. 10
An Anomalous Disease of the Tongue and Feet. By G. N. Fitch, M.D. 145
Anatomical Remembrancer, or Complete Pocket Anatomist, - 140
Antimony, Tartarized, Inoculation with, - - 157
Appointments, - - - - - - 73
Arsenic, on the Therapeutic action of. By M. Boudin, - 171
Ascites, case of, cured by injection of a stimulating Fluid in the Peritoneal cavity, - - - - - 125
Asthma, Thymic. By. M. Trousseau, - - - 165

Bibliographical Notices, - 4, 22, 37, 53, 70, 86, 101, 115, 139, 149
Bladder, chronic irritation of, caustic injections in treatment of. By Dr. Debeny, 158
Blisters in Children, - - - - 16
Blisters, the employment of, in acute diseases of the Brain. By Dr. Tritschler, 176
Blood, on the modification of, during Inflammation. By M. M. Robert Latour and Collignon, - - - 159
Bougies, of Slippery Elm Bark. By Wm. B. Herrick, M.D. - 82
Brodie's Surgical Lectures, - - - - 117
Buffalo Medical Journal, - - - - 59
Bulletin of Medical Sciences, edited by John Bell, M.D., &c. - 22

Catheterism, an easy and certain method of performing, even in the most difficult cases. By J. G. Maisoneuve, - - 15
Caustic Injections in the treatment of chronic irritation of the Bladder. By Dr. Debeny, - - - - 158
Caustics, M. Cazenave on the different sorts of, - - 93
Chemical analysis of Rhubarb or Pie Plant, by Lieut. E. R. Long, M.D. U.S.A. 78
Chemistry, Elementary, Theoretical and Practical. By Geo. Fownes, Ph. D. Edited, with additions, by Robert Bridges, M.D., 1845, - 90
Chicago, Mortality of. By W. B. Herrick, Professor of Anatomy, - 161
Chancre, Prognosis of, with reference to the probabilities of secondary symptoms. By M. Ricord, of Paris. - - - - 63
College of Physicians and Surgeons of Phila., summary of transactions of, 55, 151
Compression of the Brain, &c. By Daniel Brainard, M. D., Professor of Surgery in Rush Medical College, - - - 97
Contributions to Therapeutics. By J. Moore Nelligan, M. D. - 23
Convention, Proposed National, - - - - 136
Croton Oil and Opium, - - - - - 96, 104
Correction, - - - - - - 80
Cure of Encysted Dropsy, by exciting irritation of the Sac, - 177
Cyanosis of Infants, - - - - 110

Delirium Tremens, - - - - - - 126
Diagnosis of Fracture, - - - - - 141
Dictionary of Terms used in Medicine and the Collateral Sciences. By Richard Hoblyn, M.D. - - - - 116
Diseases of the Summer and Autumn of 1845, - - 113

PAGE

Dr. Buck on the use and abuse of Medicine, - - - 39
Domestic management of the sick room, necessary, in aid of medical treatment, for the cure of diseases. By A. Todd Thompson, M.D., F.L.S. 139
Dropsy following Scarlatina, - - - 106
Dropsy, Encysted, Cure of, by exciting irritation of the Sac, - - 177

Effects of Cinchona and the Sulphate of Quinia, - - 27
Elementary Chemistry, Theoretical and Practical. By Geo. Fownes, Ph. D. Edited, with additions, by Robert Bridges, M.D., 1845, - 90
Encephaloid Tumor of the Ovaries. By W. Butterfield, M.D., of Little Fort, Ill. 2
Endermic use of Purgatives, - - - - 141
Engrafting of Nerves, - - - - - 63
Epidemic Erysipelas, - - - - 179
Epidemic Small Pox, - - - - 174
Errata, - - - - - - 32
Erratum, - - - - - . 80
Extreme Mercurial Salivation, - - - - 127

Ferruginous Pill of Mercury, - - - - 49
Fictitious Catalogues of Medical Students, - - - 175
Fracture, on the diagnosis of. - - - 141
Fracture, on the kind of union obtained after. - - - 84

Galvanism, applied to the treatment of Uterine Hemorrhage, &c. - 29
Gout and Rheumatism,—their Pathology and Treatment, - - 122

Half-Yearly Abstract of the Medical Sciences. By W. H. Ranking, M.D. 116
Hœmostatic Means, a new.—Sheep Brains, - - - 176
Hooping Cough, - - - - - 112
Hydatids, uterine, two cases, of. By John Evans, M.D., of Attica, Ia. - 17

Influence of the shortness of the Umbilical Cord upon Labor, - 31
Inoculation with Tartarized Antimony, - - - 157
Insanity, an address on, and the establishment of a Lunatic Asylum, 10
Introductory, - - - - - - 1
Introductory Lectures, - - - - - - 9
Introductory Lecture delivered by G. S. Bedford, M.D., Prof. of Midwifery, 150
Inversion, complete, of the Uterus. By E. Fisher, M.D., - 129
Ioduret of Potassium, on the use of. in Syphilitic Affections, - - 105
Ipecacuanha, in emetic doses, a powerful restorative in some cases of exhaustion and sinking, - - - - - 111
Irritation, verminous, a direct exciting cause of diseases, not usually attributed to any of the varieties of Intestinal worms. By Dr. J. C. Scott, 132

Kermes Mineral in pulmonary affections, Clinical researches on the exhibition of, 186

Lectures on Animal Heat.—Vital Chemistry. By Thomas Spencer, M. D., Professor of the Institutes and Practice of Medicine, in Geneva Medical College, - - - - - 65
Letter from Dr. Blaney, - - - - 85
Lithontriptic Action of the Uva Ursi. By Dr. Fenolio, - - 176

Malpractice, trial for, - - - - - 51
Mammary Abscess, chronic treatment of, by the Breast Pump and Syringe, 96
Medical Convention, - - - - - - 180
Medical Students, fictitious catalogues of, - - - 175
Medical Remembrancer, or Book of Emergencies, by Edward B. S. Shaw, M. R. C. S. and L. A. S., &c. &c. - - 140
Medical Journals, - - - - - - 102
Medical Schools, - - - - - 72, 164
Mercurial Salivation, Extreme, - - - - 127
Mental Maladies.—A treatise on Insanity, &c. By Esquirol, - 70
Mercury, Ferruginous pill of, - - - - - 49
Method of ascertaining, before accouchment, whether the mother will have sufficient milk, - - - - - 32
Miss Martineau's marvellous mesmerization unmystified, - - 11
Missouri Medical and Surgical Journal, May, 1845. - - 39
Minutes of the Organization, Constitution and Ethics of the Union Medical Society of Northern Indiana, - - - 101
Modification which the blood undergoes during Inflammation. By M. M. Robert Latour and Collignon, - - - 159

PAGE

Monthly Miscellany and Journal of Health. By W. M. Cornell, M.D. - 163
Mortality of Chicago. By W. B. Herrick, M.D., Professor of Anatomy, &c. 161

Narcotic Poisoning, Vinegar in cases of, - - - 112
National Medical Convention, - - - - 96, 104
Nerves, the engrafting of, - - - - - 63
Neuralgia—introduction of Medicated fluid to the nerve. By Mr. Rynd, 75
New method of dressing wounds and ulcers. By Dr. Laugier, - 16
New Medical Society, - - - - 165
Notice to Readers and Correspondents, - - - 144

Obstetric Medicine and Surgery, Principles and Practice of. By Francis H. Ramsbotham, M.D. - - - - 115
Our Journal.—Its enlargement, - - - - 165
Ox-Gall, Remedial efficacy of, - - - 107

Pathology of Tooth Ache, - - - - 142
Phosphate of Ammonia. Its use in Gout and Rheumatism, By T. H. Buckler, M. D. - - - - 168
Placenta Prævia, on detaching the Placenta in cases of, - - 26
Placenta Prævia, The Discussion on the Treatment of. - 182
Placenta, Artificial Extraction of, before the Child, in Placental Presentations, 30
Prognosis of Chancre, with reference to the probabilities of secondary symptoms. By M. Ricord, of Paris, - - - 63
Periodical ripening and separation of the Ova of Mammifera and Man, independent of Coitus, &c. By Th. L. W. Bischoff, M.D., P.D., Giessen, 1844, - - - - - - 8
Proposed National Convention, - - - - 136
Puberty, and the critical age of Women, and the periodical discharge of the Ova, &c. By M. A. Raciborski, M.D., &c., of Paris, 1844, 4
Purgatives, Endermic use of, - - - 141
Pulmonary consumption. Frequent spontaneous cure of, and the Indications furnished by pathology for its rational treatment, - 79

Quinia, Valerianate of, - - - - 108
Quinia and Cinchona, Iodides of, - - - - 160
Quinia and Salacine, comparative merits of, - - 155
Quinine, on the use of large doses of, - - - 43, 59

Readers and Correspondents, to, - - - - 48, 64
Reduction of Dislocation of large joints, by power derived from twisted rope, 64
Retroverted Uterus, case of. By U. P. Golliday, M.D. - - 147
Report of the Commissioners of the Lunatic Asylum, or Indiana Hospital for the Insane, to the General Assembly, - - - 149
Report, the 24th, of the Bloomingdale Asylum for the Insane for 1844. By Pliny Earle, M.D. - - - - 23
Rhubarb or Pie Plant, chemical analysis of. By Lt. E. R. Long, M.D., U.S.A. 78
Rush Medical College, - - - - 48, 138

Saffron, adulteration of, - - - - 80
Salacine. The Surgeon General's Circular, - - 74
Salacine, - - - - - - 104
Salivation, Extreme Mercurial, - - - 127
Singultis, case of, cured by Acupuncture, - - - 16
Stimulants, on the use of, in inflammatory diseases of the Lungs in Children. 109
Surgery, the principles of. By John Miller, Professor of Sugery in the University of Edinburgh, &c. &c. - - - - 37
Surgical Cases, by D. Brainard, M.D., Professor of Surgery in Rush Medical College, - - - - - 20, 33, 81, 97
Surgery, the first lines of. By Samuel Cooper, - - 11
Sunlight and Health, - - - - - 173

Thymic Asthma. By M. Trousseau, - - - - 165
To the Medical Public, - - - - - 117
Tooth Ache, Pathology of, - - - - 142
Transactions of the N. Y. State Medical Society, - - - 86
Tubercle of the Lungs in Children, a tabular view of the seat of, &c. 90

Umbilical Cord, influence of shortness of, on Labor, - - 31
Urinary and Sexual organs, anatomy of. By G. J. Guthrie, F. R. S. - 53

PAGE

Use and Abuse of Medicine. By Dr. Buck, - - - 39
Use of Ioduret of Potassium in Syphilitic affections, - - 105
Uterus, case of retroverted, - - - - 147
Uva Ursi, Its Lithontripic action, - - - - 176

Vinegar, in cases of narcotic poisoning, - - - 112
Vital Chemistry, &c., by Thomas Spencer, M. D., Prof. &c. in Med. Institute of Geneva College, Review of, - - - 65
Verminous Irritation, a direct exciting cause of diseases not usually attributed to any of the varieties of Intestinal Worms, - - 132

Wounds and Ulcers, new method of dressing. By Dr. Laugier, - 16

Cont'd as Ill. & Indiana
M & S Journ.

### PROSPECTUS

OF THE

### ILLINOIS & INDIANA

### MEDICAL & SURGICAL JOURNAL.

THE continuation of the Illinois Medical & Surgical Journal, which has now completed its 2d volume, will hereafter be published with or under the above title. It will appear as early as practicable in April next, and thereafter on the 10th of every other month in an entirely new form, much enlarged and improved, and will be issued simultaneously in the cities of Chicago and Indianapolis. Each number will contain 96 pages octavo, on good paper, with a neat cover and excellent typographical execution. The yearly volume of six numbers will thus comprise 576 pages exclusive of cover and advertisements. The editorial conduct of the work will be divided among the following persons,

DR'S BLANEY, BRAINARD, & HERRICK of Chicago, and
DR. JOHN EVANS of Indianapolis.

The contents of each number will be arranged as follows:

Original communications—Reviews of New Works—Bibliographial notices—Editorial Department and General Intelligence—Report on the Progress of some one of the branches of Medical Science—Selections from Foreign and Domestic Journals.

Arrangements have been made by which it is anticipated that the *Original* department of the Journal will be well sustained. A number of distinguished and accomplished physicians are expected to render their assistance as co-laborers.

Reviews of New Works will be as numerous as the importance of the issues from the medical press, may renderad visable, and will be characterized by fairness and impartiality.

Under Bibliographical Notices, readers will be informed of the publication of all new and valuable books on Medical science, and enabled to judge of their respective merits.

In the Editorial Department it will be endeavored to discuss all subjects of interest to the profession, and place them before the readers of the journal, in the clearest light possible.

It is also contemplated that each number shall contain a report or abstract of the advancement of some individual department of Medical science.

An extensive exchange list and other resources, will enable the Editors to place before subscribers at an early date, important improvements in all departments, as reported in numerous Domestic and Foreign Journals.

The Journal, thus enlarged and improved, will, it is believed, assert for itself its claims upon the co-operation of the profession in this North Western section of our country. The increased expenses which the publishers will be obliged to assume to render the Journal worthy of a wide circulation, demands as a recompense an extentension of their subscription list. They now present, in the enlarged series, *three* times the amount of reading matter for *double* the price of the former subscription. The continuation of the Journal, however, they may remark, is rendered independent of the increase in their present list. They, however, confidantly trust, that the generosity of the Medical public, for whose benefit this increase of expenditure and responsibility is assumed, will, by their patronage indemnify them against pecuniary loss.

### TERMS OF SUBSCRIPTION.

The *Illinois and Indiana Medical and Surgical Journal* will be sent to single subscribers, in its enlarged form, for $2,00 per annum, *always in advance*, and to every subscriber sending in the amount of his subscription before the issue of the 2d No., will be forwarded the last Vol. (Vol. II.) free of charge.

As an inducement to Postmasters and others to act as agents, three copies will be sent to any desired address upon the receipt of $5,00. All monies to be forwarded to the Senior Editor, free of expense.

Chicago, March 17, 1846.

